# A Study of the Clinical Profile of Iron Deficiency Anemia in Children With Sickle Cell Disease in a Tertiary Care Center

**DOI:** 10.7759/cureus.70087

**Published:** 2024-09-24

**Authors:** Naramreddy Sudheesh Reddy, Keta Vagha, Ashish Varma, Chaitanya Kumar Javvaji

**Affiliations:** 1 Pediatrics, Jawaharlal Nehru Medical College, Datta Meghe Institute of Higher Education and Research, Wardha, IND

**Keywords:** cariology, folic acid deficiency, iron deficiency, organomegaly, sickle cell disease

## Abstract

Background

Sickle cell disease (SCD) and iron deficiency anemia (IDA) are significant global health concerns, particularly in pediatric populations. This study investigates the prevalence, clinical impact, and management challenges of IDA in children with SCD.

Methods

A prospective observational study was conducted at the Department of Pediatrics, Jawaharlal Nehru Medical College* *(JNMC) and Acharya Vinoba Bhave Rural Hospital (AVBRH), Sawangi, from June 2022 to May 2024. The study included 60 children diagnosed with SCD. Comprehensive assessments were performed, including medical histories, physical examinations, and hematological investigations. Diagnosis of IDA was based on hemoglobin levels, mean corpuscular volume (MCV), red cell distribution width (RDW), peripheral blood smears, serum iron levels, and serum ferritin concentrations.

Results

Of the 60 participants, 15% exhibited iron deficiency. No significant gender differences were found in iron deficiency status. A significant association was observed between SCD type and the presence of pallor (p = 0.025) and sickle cell crises (p = 0.023). The study also found no significant association between SCD type and the presence of organomegaly (p = 0.079) or iron deficiency status (p = 0.675). The mean hemoglobin levels varied across SCD types, with sickle cell anemia patients showing lower levels than those with sickle cell trait or disease.

Conclusion

Diagnosing and managing IDA in children with SCD is complex due to overlapping hematological features and the risk of iron overload from frequent transfusions. Tailored diagnostic and therapeutic approaches are essential to improving hemoglobin levels, reducing complications, and enhancing the quality of life for affected children. This study provides valuable insights for refining clinical practices and emphasizes the need for a multidisciplinary approach to managing these intertwined conditions.

## Introduction

Hemoglobin S is the outcome of a mutation in the β-globin gene, which causes a set of inherited red blood cell diseases known as sickle cell disease (SCD). Because of the decreased blood flow caused by this aberrant hemoglobin, red blood cells become stiff and sickle-shaped, which can result in several issues. Over 300,000 babies worldwide are affected by SCD each year [1].

Iron deficiency anemia (IDA) is characterized by reduced red blood cell production due to insufficient iron, which is essential for hemoglobin synthesis. IDA results from inadequate dietary intake, increased iron requirements during growth or pregnancy, chronic blood loss, and impaired absorption [2]. Increased iron requirements during certain life stages contribute to IDA. Children and adolescents experience rapid growth spurts that increase the demand for iron to support expanded blood volume and muscle mass. Similarly, pregnant women require additional iron to support the growing fetus and placenta and to accommodate increased blood volume. These periods of rapid growth and physiological changes elevate the body's need for iron, making these groups particularly susceptible to IDA [3].

Chronic blood loss is a significant contributor to IDA. Women of reproductive age are particularly susceptible to IDA due to regular menstrual blood loss. Gastrointestinal bleeding from conditions such as peptic ulcers, gastritis, hemorrhoids, or colorectal cancer can also lead to chronic blood loss and, subsequently, IDA. Additionally, prolonged use of nonsteroidal anti-inflammatory drugs can cause gastrointestinal bleeding, further aggravating iron depletion [4]. Impaired iron absorption is another significant factor in the development of IDA. Gastrointestinal disorders such as celiac disease, Crohn's disease, and ulcerative colitis can damage the intestinal lining, hindering iron absorption. Surgical procedures that alter the gastrointestinal tract, such as bariatric surgery, can also decrease iron absorption. Certain medications and substances can also interfere with iron absorption; for instance, antacids and proton pump inhibitors reduce stomach acid, which is essential for the absorption of non-heme iron [5].

General prevalence and impact on pediatric population

IDA affects a significant portion of the global population, particularly in developing countries with more prevalent nutritional deficiencies. IDA is especially concerning in pediatric populations due to its potential to impair cognitive and physical development. The World Health Organization estimates that iron deficiency is the primary cause of anemia in approximately 42% of children under five worldwide. The impact of IDA in children includes delayed psychomotor development, behavioral disturbances, and increased susceptibility to infections, which underscores the importance of early diagnosis and treatment [6].

Challenges in diagnosing and managing IDA in children with SCD

Diagnosing IDA in children with SCD is challenging due to overlapping hematological features. SCD causes chronic hemolytic anemia, resulting in normocytic or macrocytic red blood cells, which can obscure the microcytic and hypochromic anemia typical of IDA. Frequent blood transfusions in SCD patients can lead to iron overload, complicating the assessment of iron status as it masks IDA symptoms. Elevated ferritin levels due to inflammation, iron overload, or liver damage further obscure diagnosis [7]. Due to ongoing hemolysis and chronic inflammation, children with SCD often have altered serum transferrin and low hepcidin levels, affecting traditional iron studies such as serum iron, total iron-binding capacity, and transferrin saturation. Accurate diagnosis of IDA in SCD requires a comprehensive evaluation, including detailed hematological mean corpuscular volume (MCV) and red cell distribution width (RDW) and iron studies such as serum ferritin, transferrin saturation, alongside newer markers such as reticulocyte hemoglobin content and soluble transferrin receptor. Clinical history, dietary assessments, and transfusion frequency should also be considered [8].

Potential complications and clinical significance of concurrent SCD and IDA

The coexistence of IDA and SCD exacerbates morbidity and complicates clinical management. SCD, characterized by chronic hemolytic anemia and vaso-occlusive crises (VOC), compromises red blood cell oxygen-carrying capacity. IDA further diminishes this capacity, leading to more severe anemia and potentially increasing the frequency and severity of painful VOC, which can result in complications such as acute chest syndrome, stroke, and organ damage [9]. Additionally, iron deficiency impairs immune function, making children with SCD more susceptible to infections due to compromised cell-mediated immunity and reduced pro-inflammatory cytokine production. This is especially concerning as SCD patients are already at high risk for infections, which can trigger acute crises and are a leading cause of morbidity and mortality in this population [10].

Addressing IDA in children with SCD is crucial for improving hemoglobin levels, enhancing immune function, and reducing infection risk. This can lower the frequency and severity of VOC and improve overall quality of life. Managing IDA requires a multidisciplinary approach, balancing iron replenishment with the risk of iron overload from blood transfusions. Tailored treatment strategies, including monitoring iron levels and using iron chelation therapy when necessary, alongside nutritional interventions and dietary education, are essential [11].

This study aims to investigate the prevalence, clinical impact, and management challenges of IDA in children with SCD. It seeks to identify the frequency of concurrent IDA in pediatric SCD patients, assess health outcomes, elucidate risk factors for IDA, and evaluate current diagnostic and treatment strategies. The goal is to provide insights to improve clinical practices and enhance the management and quality of life for children suffering from SCD and IDA.

## Materials and methods

Study design, setting, and study period

A prospective observational study design was employed, enabling systematic observation and documentation of participants' characteristics, experiences, and outcomes over time to explore relationships between variables. The present observational study was conducted at the Department of Pediatrics, Acharya Vinoba Bhave Rural Hospital (AVBRH), Sawangi, after obtaining clearance from the institutional ethics committee. Two years, from June 2022 to May 2024, were dedicated to data collection.

Study participants

Children diagnosed with SCD were the focus of the study, reflecting the specific demographic of interest due to the prevalence and health implications associated with the condition.

Ethics consideration and sample size

The Institutional Review Board of Datta Meghe Institute of Higher Education and Research in Wardha has given its approval to the research study's protocol, which is designated by the number DMIMS(DU)/IEC/2022/1072. The sample size for the study was determined using the following formula: n = (DEFF*Np(1-p))/((d2/Z21-α/2*(N-1)+p*(1-p)), where N represents the size of the population (taking into account the finite population correction factor), p is the hypothesized percentage of the outcome factor in the population, d stands for the confidence limits as a percentage of 100 (expressed as an absolute value of +/-%), and DEFF signifies the design effect. A total of 60 cases were included in this study.

Study procedure

Following the ethical approval from the Institutional Ethical Committee, the purpose and objectives of the study were explained to the parents or caregivers who fulfilled the selection criteria. The informed consent form provided comprehensive information regarding the study, ensuring transparency and adherence to ethical guidelines. All the cases of hemoglobinopathy, including sickle cell anemia, sickle cell trait, and heterogenous hemoglobinopathy, were included in the study. Patients who are already on iron supplementation and who are not willing to give consent are excluded from the study. Subsequently, the enrolled children underwent a comprehensive assessment process, which included the collection of detailed medical histories, thorough physical examinations, and a series of investigative procedures to establish complete clinical profiles. All gathered information was meticulously recorded in a structured proforma to ensure systematic documentation and analysis. Diagnosis of IDA was conducted by qualified pediatricians prior to the inclusion of participants in the study. This diagnostic process relied on a multifaceted approach, taking into account various factors such as medical history, observed signs like pallor and koilonychia, and results from a range of investigative tests. These investigations encompassed a battery of hematological parameters, including hemoglobin levels, MCV, mean corpuscular hemoglobin concentration, mean corpuscular hemoglobin (MCH), and RDW. Additionally, peripheral blood smears were examined for morphological abnormalities indicative of anemia, such as microcytic and hypochromic features, and the presence of anisocytosis and poikilocytosis.

Furthermore, serum iron levels, total iron-binding capacity, and serum ferritin concentrations were assessed to provide insights into the iron status of the participants. Criteria for defining IDA were established based on predetermined thresholds, including hemoglobin levels below 11 mg/dL, MCV measurements less than 80 fL, and RDW values exceeding 14.5%. Peripheral blood smears showing characteristic morphological changes consistent with IDA also contributed to the diagnostic process. Additionally, serum iron levels below 50 mcg/dL and serum ferritin concentrations below 30 ng/mL, in conjunction with positive C-reactive protein levels exceeding 5 mg/dL, further supported the diagnosis of IDA in eligible participants.

Statistical analysis

After the data collection process was completed, the statistical analysis was conducted to investigate the relationships between variables and meet the research objectives. The data were inputted into a secure database, and any errors were corrected. Descriptive statistics were used to summarize the study population and key variables, while both descriptive and inferential statistical analyses were performed using Stata software (StataCorp. 2007. Stata Statistical Software: Release 10. College Station, TX: StataCorp LLC). The Chi-square or Fisher's exact test was used to examine associations for categorical data, and the Student's t-test and ANOVA were employed to compare means for continuous data. A significance level of less than 0.05 was used to interpret the results based on p-values. Effect size measures were also calculated to quantify the strength and direction of associations, providing insights into the practical significance of the relationships. This analysis provided valuable insights into the prevalence, associations, and predictors of IDA in children with SCD, aiding in the understanding and management of the condition.

## Results

Table 1 displays the status of iron deficiency, capturing the frequency and percentage distribution of individuals affected. Within the dataset, the absence of iron deficiency was predominant, with 51 cases constituting 85% of the total sample of 60 participants. Conversely, a smaller proportion, comprising nine cases or 15% of the population, exhibited signs of iron deficiency. The cumulative percentages illustrated a comprehensive view, showing that 85% of the participants were not afflicted with iron deficiency, while the remaining 15% were. In total, the table accounted for 60 individuals, representing the entirety of the surveyed population.

**Table 1:** Status of iron deficiency in sickle hemoglobinopathy

Status of Iron Deficiency	Frequency	Percent (%)
Yes	9	15
No	51	85
Total	60	100

Table 2 delineates the status of iron deficiency with respect to gender, outlining the frequency and percentage distribution for each subgroup. Among individuals unaffected by iron deficiency, 27 males and 24 females were recorded, constituting 84.38% and 85.71% of their respective gender groups and accounting for a total of 51 cases. Conversely, among those afflicted with iron deficiency, five males and four females were identified, representing 15.63% and 14.29% of their respective gender categories, totaling nine cases. The cumulative percentages for each gender category showcased the distribution of iron deficiency status within the male and female cohorts. In total, the table encompassed 32 males and 28 females, with each gender group contributing 100% to the overall sample size of 60 individuals. The statistical analysis indicated a Pearson chi-square value of 0.0210 with a p-value of 0.885, suggesting no significant association between gender and iron deficiency status.

**Table 2:** Status of iron deficiency by gender among the study subjects

Status of Iron Deficiency	Male (%)	Female (%)	Total (%)	Pearson Chi-Square Value	P-value
Present	5 (15.63)	4 (14.29)	9 (15)	0.0210	0.885
Absent	27 (84.38)	24 (85.71)	51 (85)		
Total	32	28	60		

Among individuals diagnosed with sickle cell anemia, 20 exhibited organomegaly while 14 did not, constituting 50% and 70% of their respective categories and accounting for a total of 34 cases. For those diagnosed with SCD, one individual displayed organomegaly while two did not, representing 2.5% and 10% of their respective groups, totaling three cases. Individuals with sickle cell trait were observed to have 19 cases with organomegaly and four cases without, comprising 47.5% and 20% of their respective categories and making up a total of 23 cases.

Table 3 depicts the association between sickle cell crisis occurrence and the type of sickle cell. Overall, the table encompassed 50 cases of sickle cell crisis and 10 cases without, with each category contributing 100% to the total sample size of 60 individuals. The statistical analysis revealed a Pearson chi-square value of 7.5529 with a corresponding p-value of 0.023, indicating a significant association between sickle cell type and the occurrence of sickle cell crisis.

**Table 3:** Association between sickle cell crisis occurrence and the type of sickle cell

Type of Sickle Cell	Sickle Cell Crisis Absent (%)	Sickle Cell Crisis Present (%)	Total	Pearson Chi-Square Value	P-value
Sickle cell anemia	9 (90)	25 (50)	34 (56.67)	7.5529	0.023
Sickle cell disease	1 (10)	2 (4)	3 (5)		
Sickle cell trait	0	23(46)	23(38.33)		
Total	10	50	60		

Among individuals diagnosed with sickle cell anemia, the mean hemoglobin level was 8.46 g/dL, with a standard deviation of 2.91, based on 34 observations. For those diagnosed with SCD, the mean hemoglobin level was 7.73 g/dL, with a standard deviation of 2.73, derived from three observations. Individuals with sickle cell trait exhibited a mean hemoglobin level of 10.45 g/dL, with a standard deviation of 2.47, based on 23 observations. The total mean hemoglobin level across all sickle cell types was calculated to be 9.19 g/dL, with a standard deviation of 2.88, encompassing 60 observations. Table 4 provides an analysis of hemoglobin levels across different types of sickle cell disorders, presenting the mean hemoglobin, standard deviation, and frequency for each group.

**Table 4:** Analysis of hemoglobin levels across different types of sickle cell disorders

Type of Sickle Cell	Mean Hemoglobin(mg/dL)	Standard Deviation	Frequency
Sickle cell anemia	8.46	2.91	34
Sickle cell disease	7.73	2.73	3
Sickle cell trait	10.45	2.47	23
Total	9.19	2.88	60

The analysis of sickle hemoglobinopathy categorized by serum iron levels showed that sickle cell anemia exhibited a mean serum iron level of 78.41, with a standard deviation of 39.94, observed 34 times within the sample. SCD displayed a mean of 109.00, with a standard deviation of 54.03, occurring three times. Sickle cell trait demonstrated a mean of 91.78, with a standard deviation of 56.54, present in 23 instances. The cumulative statistics for mean, standard deviation, and frequency across all types were also provided.

Table 5, detailing the distribution of sickle hemoglobinopathy by serum ferritin, presents a comprehensive breakdown of the data, encompassing three main types of sickle cell conditions: sickle cell anemia, SCD, and sickle cell trait. The table depicted the mean, standard deviation, and frequency for each type, providing valuable insights into their distribution within the sample population. Sickle cell anemia exhibited a mean serum ferritin level of 102.07 with a standard deviation of 123.16, occurring 34 times within the dataset. SCD, comparatively less prevalent, displayed a mean of 156.00 with a standard deviation of 132.38, found only three times. On the other hand, the sickle cell trait showcased a mean of 93.23 with a standard deviation of 77.15, occurring 23 times. The overall statistics for mean, standard deviation, and frequency were also presented, summarizing the data across all types.

**Table 5:** Distribution of sickle hemoglobinopathy by serum ferritin

Type of Sickle Cell	Mean	Standard Deviation	Frequency
Sickle cell anemia	102.07	123.16	34
Sickle cell disease	156.00	132.38	3
Sickle cell trait	93.23	77.15	23
Total	101.38	107.12	60

Among individuals diagnosed with sickle cell anemia, 28 were identified as not having an iron deficiency, while six had iron deficiency, totaling 34 observations. This accounts for 54.90% and 66.67% of the respective categories. For individuals with SCD, three individuals were observed without iron deficiency, constituting 5.88% of this category, while no individuals with iron deficiency were observed. For sickle cell trait, 20 individuals were noted without iron deficiency, while three individuals had iron deficiency, resulting in a total of 23 observations. The total number of individuals without iron deficiency was 51, while the number with iron deficiency was nine, summing up to 60 observations. Pearson's chi-square test yielded a statistic of 0.7853 with a p-value of 0.675, indicating no significant association between sickle cell crisis and the status of iron deficiency across the different sickle cell types (Table 6).

**Table 6:** Type of sickle hemoglobinopathy and status of iron deficiency

Type of Sickle Cell	Iron Deficiency Present	Iron Deficiency Absent	Total	Pearson Chi-Square Test	P-value
Sickle cell anemia	6 (66.67)	28 (54.9)	34 (56.67)	0.7853	0.675
Sickle cell disease	0	3 (5.88)	3 (5)		
Sickle cell trait	3 (33.33)	20 (39.22)	23 (38.33)		
Total	9	51	60		

In the MCV <80 category, there were 20 instances of sickle cell anemia, 0 cases of SCD, and 15 occurrences of sickle cell trait, totaling 35 instances. This accounted for 57.14% of sickle cell anemia, 0.00% of SCD, and 42.86% of sickle cell trait within the sample. For MCV >80, there were 14 cases of sickle cell anemia, three instances of SCD, and eight occurrences of sickle cell trait, amounting to 56.00%, 12.00%, and 32.00%, respectively, making up a total of 25 cases. The grand total of cases across all MCV interpretations stood at 34 for sickle cell anemia, three for SCD, and 23 for sickle cell trait, summing up to 60 instances. The Pearson chi-square test yielded a value of 4.6518 with a significance probability of 0.098, indicating the degree of association between MCV interpretation and the type of sickle hemoglobinopathy. Table 7 illustrates the distribution of MCV interpretations with respect to the type of sickle hemoglobinopathy.

**Table 7:** Distribution of MCV interpretations with respect to the type of sickle hemoglobinopathy MCV: mean corpuscular volume

Type of Sickle Cell	MCV <80 (%)	MCV >80 (%)	Total (%)	Pearson Chi-Square Test	Significance Probability
Sickle cell anemia	20 (57.14)	14 (56)	34 (56.67)	4.6518	0.098
Sickle cell disease	0	3 (12)	3 (5)		
Sickle cell trait	15 (42.86)	8 (32)	23 (38.33)		
Total	35	25	60		

In the 24-30 MCH interpretation category, there were 20 instances of sickle cell anemia, three cases of SCD, and 10 occurrences of sickle cell trait, totaling 33 instances. This accounted for 58.82% of sickle cell anemia, 100.00% of SCD, and 43.48% of sickle cell trait within the sample. For MCH values falling outside the 24-30 range, there were 14 cases of sickle cell anemia and 13 instances of sickle cell trait, amounting to 41.18% and 56.52%, respectively, making up a total of 27 cases. The grand total of cases across all MCH interpretations stood at 34 for sickle cell anemia, three for SCD, and 23 for sickle cell trait, summing up to 60 instances. The Pearson chi-square test yielded a value of 3.8890 with a significance probability of 0.143, indicating the degree of association between MCH interpretation and the type of sickle hemoglobinopathy.

## Discussion

Status of iron deficiency in sickle hemoglobinopathy

A total of 51 individuals (85.00%) did not have iron deficiency, while nine individuals (15.00%) did, out of 60 surveyed individuals. There were 27 males (84.38%) and 24 females (85.71%) who did not have iron deficiency. Conversely, five males (15.63%) and four females (14.29%) did, with a sample size of 60 individuals. A study by Kim et al. evaluated the diagnostic value of reticulocyte-hemoglobin equivalent and found that traditional red blood cell indices were more accurate in detecting IDA in SCD children than newer biomarkers such as reticulocyte-hemoglobin equivalent, which is less affected by inflammation [12]. This suggests that standard hematological parameters remain valuable in assessing iron status in this population.

Patel et al. examined the iron status of 180 SCD patients and found that while most were iron-sufficient, a significant minority exhibited either iron deficiency or overload [13]. This study emphasized the need for careful assessment of iron status before initiating treatment, given the varying iron storage levels among SCD patients. Furthermore, a study conducted in Brazzaville highlighted that 35.9% of children with homozygous SCD had iron overload, and 4.8% had iron deficiency. The study also identified factors such as infections and blood transfusions contributing to iron metabolism abnormalities [14]. A Nigerian study by Akodu et al. determined specific red cell indices cutoffs for detecting iron deficiency in SCD children, revealing that standard reference values used for the general population may not apply to SCD patients. They proposed specific cutoff values for MCV and MCH that showed better diagnostic performance in SCD [15].

Type of sickle hemoglobinopathy by pallor

Among those with sickle cell anemia, 21 (72.41%) had pallor and 13 (41.94%) did not. For SCD, two (6.90%) had pallor, and one (3.23%) did not. Among those with sickle cell trait, six (20.69%) had pallor, and 17 (54.84%) did not. The Pearson chi-square value of 7.4181 with a p-value of 0.025 indicates a significant association between sickle cell type and the presence of pallor. Majumder et al. documented two cases of SCD presenting with pallor and recurrent bone and joint pain, underscoring the clinical significance of pallor as a symptom associated with severe manifestations of the disease [16]. Their cases were managed with hydroxyurea to reduce acute chest syndrome episodes and pain crises, highlighting the therapeutic challenges in managing pallor in SCD patients.

McClain and Kain discussed the role of pediatric palliative care in SCD, emphasizing the importance of early intervention and symptom management, including pallor [17]. They noted that effective palliative care can improve the quality of life for SCD patients by addressing chronic symptoms and complications. Darko et al. explored gallstones in Ghanaian children with SCD and found a significant prevalence of pallor, linking it to hemolysis and other complications [18]. This study reinforces the need for comprehensive clinical evaluation in SCD patients presenting with pallor to effectively detect and manage underlying conditions. Nwogu-Onyemkpa et al. evaluated inpatient palliative care use among adult SCD patients, revealing low utilization despite significant morbidity associated with pallor and other symptoms [19]. This study suggests the need for more integrated palliative care services to manage symptoms such as pallor in pediatric and adult SCD populations.

Type of sickle hemoglobinopathy by sickle cell crisis

A total of 25 individuals (90.00%) with sickle cell anemia experienced a crisis, while nine (50.00%) did not. For SCD, one (10.00%) experienced a crisis, and two (4.00%) did not. None of those with sickle cell trait experienced a crisis, while 23 (46.00%) did not. The Pearson chi-square value of 7.5529 with a p-value of 0.023 indicates a significant association between sickle cell type and the occurrence of sickle cell crisis. Vuong et al. investigated the impact of hospital admissions for VOC on health-related quality of life (HRQoL) in children with SCD [20]. They found that hospitalizations for VOC significantly decreased HRQoL, particularly affecting physical functioning and school performance up to 12 months after hospitalization.

Allali et al. explored the potential of sputum interleukin 6 (IL-6) levels as a predictor for acute chest syndrome during VOC in children with SCD. Their study highlighted that elevated IL-6 levels were associated with a higher risk of developing acute chest syndrome, indicating the importance of early markers in managing crises [21]. Almahmoud et al. reviewed emergency department management of fever and acute painful crises in children with SCD [22]. They emphasized that timely administering fluids, analgesia, and antibiotics is crucial in managing VOC and preventing complications.

Type of sickle hemoglobinopathy by serum iron

The following were the mean serum iron levels: 78.41 µg/dL for sickle cell anemia, 109.00 µg/dL for SCD, and 91.78 µg/dL for sickle cell trait, with an overall mean of 85.07 µg/dL. The ANOVA F-statistic of 0.94 with a p-value of 0.3954 indicates no significant differences in the mean serum iron levels among the types. Bartlett's test suggests no significant difference in variances (chi-square = 3.0643, p-value = 0.216). Oyiro et al. assessed serum ferritin as a marker of iron levels in patients with sickle cell anemia at Kenyatta National Hospital [23]. They found that 70.5% of their subjects had elevated serum ferritin levels, primarily due to frequent blood transfusions and chronic hemolysis. This study emphasizes the risk of iron overload in SCD patients receiving multiple transfusions. Makulo et al. conducted a study on Congolese children with steady-state sickle cell anemia and found that 21.4% had elevated iron stores [24]. The primary risk factor identified was the history of poly transfusions, further highlighting the association between transfusion practices and iron overload in SCD patients. Olufemi et al. explored iron overload in non-chronically transfused preschool children with sickle cell anemia. They reported that 14.4% of their subjects had elevated serum ferritin levels, indicating that even children not receiving regular transfusions can be at risk for iron overload due to chronic hemolysis [25].

Type of sickle hemoglobinopathy by serum ferritin

The following were the mean serum ferritin levels: 102.07 ng/mL for sickle cell anemia, 156.00 ng/mL for SCD, and 93.23 ng/mL for sickle cell trait, with an overall mean of 101.38 ng/mL. The ANOVA F-statistic of 0.45 with a p-value of 0.6408 indicates no significant differences in the mean serum ferritin levels among the types. Bartlett's test suggests no significant difference in variances (chi-square = 4.9987, p-value = 0.082). Oyiro et al. conducted a study at Kenyatta National Hospital, finding that the mean serum ferritin was significantly elevated in 70.5% of patients with sickle cell anemia [23]. They concluded that high serum ferritin levels are significantly associated with the number of blood transfusions received, emphasizing the need for annual serum ferritin measurements to manage iron status.

Makulo et al. studied Congolese children with SCD and found that 21.4% had elevated iron stores, primarily linked to frequent blood transfusions [24]. This study highlighted the prevalence of hyperferritinemia in SCD patients and the necessity for regular monitoring to prevent iron overload. Olufemi et al. evaluated iron overload in non-chronically blood-transfused preschool children with SCD. They reported that 20.6% of subjects had iron overload, stressing the importance of regular assessment of serum ferritin even in the absence of frequent transfusions [25].

Type of sickle hemoglobinopathy and status of iron deficiency

A total of 28 individuals with sickle cell anemia were iron deficient (54.90%), three with SCD (5.88%), and 20 with sickle cell trait (39.22%). Among those with iron deficiency, six had sickle cell anemia (66.67%), none had SCD (0.00%), and three had sickle cell trait (33.33%). The Pearson chi-square value of 0.7853 with a p-value of 0.675 indicates no significant association between sickle cell type and iron deficiency. Ofosu et al. evaluated the diagnostic value of reticulocyte hemoglobin equivalent among children with SCD [26]. While reticulocyte hemoglobin equivalent provides a more real-time assessment of iron status unaffected by inflammation, red cell indices such as MCV and MCH were more accurate in detecting iron deficiency in these patients.

Patel et al. conducted a study in Western India and concluded that while most sickle cell anemia patients were iron-sufficient, a significant number exhibited either iron deficiency or iron overload [26]. This study emphasizes the need for careful assessment of iron status before administering iron therapy to sickle cell anemia patients. Atipo-Ibara Ollandzobo et al. reported that 4.8% of children with homozygous SCD in Brazzaville had iron deficiency [14]. This study highlighted that iron metabolism abnormalities are relatively frequent in SCD patients, justifying regular monitoring during follow-up.

MCV interpretation vs. the type of sickle hemoglobinopathy

The following were the MCV interpretations: MCV <80 includes 20 cases of sickle cell anemia (57.14%), 0 of SCD (0.00%), and 15 of sickle cell trait (42.86%). MCV >80 includes 14 cases of sickle cell anemia (56.00%), three of SCD (12.00%), and eight of sickle cell trait (32.00%). The Pearson chi-square value of 4.6518 with a p-value of 0.098 indicates no significant association between MCV interpretation and sickle hemoglobinopathy. Pradhan et al. low-dose hydroxyurea therapy improved hematological parameters, including MCV, in SCD patients. Their study showed hydroxyurea increased MCV, indicating better disease control and reduced hemolysis [27]. Kuo et al. highlighted the importance of monitoring MCV in SCD patients undergoing extracorporeal membrane oxygenation. They noted that higher MCV values were associated with improved oxygenation and better clinical outcomes [28].

MCH interpretation vs. the type of sickle hemoglobinopathy

The following were the MCH interpretations: MCH 24-30 includes 20 cases of sickle cell anemia (58.82%), three of SCD (100.00%), and 10 of sickle cell trait (43.48%). For MCH <24 or >30, there are 14 cases of sickle cell anemia (41.18%), 0 of SCD (0.00%), and 13 of sickle cell trait (56.52%). The Pearson chi-square value of 3.8890 with a p-value of 0.143 indicates no significant association between MCH interpretation and sickle hemoglobinopathy. Lykavieris et al. investigated autoimmune liver disease in children with SCD and highlighted the impact of chronic hemolytic anemia on liver function, which can be indirectly associated with variations in MCH levels due to the chronic hemolysis characteristic of SCD [29]. Dougherty et al. examined muscle strength and power deficits in children with type SS SCD and found that lower MCH values were often associated with more severe anemia and related complications, suggesting the importance of monitoring MCH in these patients [30].

Limitations of the study

While adequate for initial observations, the study's sample size of 60 participants may not be representative of the broader population of children with SCD. Larger studies are needed to validate these findings and provide more generalizable results. Conducted at a single medical center, the study's findings may be influenced by local practices and patient demographics, potentially limiting the generalizability of the results to other settings and regions. The two-year study duration, while sufficient for initial observations, may not capture long-term trends and outcomes related to IDA in SCD. Longer follow-up periods are necessary to understand the chronic impacts and progression of these conditions.

## Conclusions

The study highlights the significant challenges in diagnosing and managing IDA in children with SCD. Despite 85% of the cohort not having an iron deficiency, the 15% prevalence indicates the need for careful monitoring. The study found no significant gender differences in iron deficiency status but noted a significant association between SCD type and the presence of pallor and sickle cell crises. These findings emphasize the need for tailored diagnostic and treatment strategies to manage concurrent IDA and SCD effectively, aiming to improve clinical outcomes and quality of life for affected children.
